# Improving the effective maternal-child health care coverage through synergies between supply and demand-side interventions: evidence from Mexico

**DOI:** 10.7189/jogh.09.020433

**Published:** 2019-12

**Authors:** Edson Serván-Mori, Diego Cerecero-García, Ileana B Heredia-Pi, Carlos Pineda-Antúnez, Sandra G Sosa-Rubí, Gustavo Nigenda

**Affiliations:** 1 Center for Health Systems Research. National Institute of Public Health, Cuernavaca, Morelos, Mexico; 2 National School of Nursing and Obstetrics. National Autonomous University of Mexico, Mexico City, Mexico

## Abstract

**Background:**

Over the last two decades, the Mexican government has released several efforts to achieve universal health coverage (UHC), based on the principles of fairness and social protection, to reduce the inequities in utilization, access, and quality of care existing in the health system. Two of the most important social public policies that have targeted the population without access to social security include the 1997 conditional cash transfers (CCT) program known as Prospera (formerly Oportunidades or Progresa) and the Seguro Popular de Salud (SPS by its Spanish initials), launched in 2003. These two programs, so far, have survived changes in the federal administrations being the most longstanding social programs targeting poor (or unprotected) populations ever in the history of modern Mexico. We tested the existence of positive synergies between demand-side (or CCT-Prospera) and supply-side (or Seguro Popular de Salud, SPS) social programs in the achievement of effective coverage (EC) of maternal-child health interventions in Mexico.

**Methods:**

We performed a retrospective-cohort analysis to 6413 women aged 12-49 years who participated in a probabilistic survey conducted in 2012. We calculated EC as the product of three indexes: need, utilization and quality of health care. Correlates of EC were identified estimating a logistic regression model. We also presented adjusted EC by specific women groups.

**Results:**

EC among beneficiaries of both programs was similar to estimates in Social Security affiliates (54%). For those not affiliated to any of the programs or those who received benefits for only one of them, the EC was 47.6% and 45.5% respectively. Adjusted estimates of EC suggest that overall, having both programs (Prospera + SPS) has a positive effect on maternal and child care coverage, which makes the observed differences in EC not statistically significant between those affiliated to both programs in comparison with the observed in the population with social security.

**Conclusions:**

Results support positive synergies between Prospera and SPS in the reduction of the gaps in EC. The most vulnerable population groups need to be reached by the combination of these programs so that public health efforts translate into greater EC of maternal health services and better maternal-child outcomes.

Despite extensive policy efforts to implement social policy programs, aligned to international initiatives, such as the signing of the MDGs, in Mexico, maternal health remains a pressing public health issue, particularly among poor and uninsured populations [1-4].

Monitoring the performance of health systems is fundamental to guide effective policies in order to secure the universal right to health care [5]. One metric used to evaluate the performance of the health system is the effective coverage (EC) of health interventions. EC diverges from the traditional way of measuring coverage for health interventions by measuring the fraction of potential gain in health that a health system can provide through an available intervention [6]. It is a health metric that allows a comprehensive evaluation of the performance of health systems, combining coverage, access, use and efficacy of health interventions [5,7]. There are no recent studies that document the achievements in terms of EC for maternal and child health interventions in Mexico.

Among the initiatives implemented in the international context to improve access to health services and reduce the risk for households to fall into Out-of-Pocket Expenditures on Healthcare (OOPE), as well as increasing productivity and reducing wage loss, interventions have been designed to support health financing – both, on the demand and supply sides. Examples of the former are the delivery of coupons, subsidies and conditional cash transfers (CCT) that promote changes in risk behaviors in the target population and incentives for the use of health services. Health insurance schemes for larger population groups and pay-for-performance programs for health providers are examples of interventions on the supply side [8-10].

Over the last two decades, the Mexican government has released several efforts to achieve UHC, based on the principles of fairness and social protection, to reduce the inequities in utilization, access, and quality of care that exist in the health system [11,12]. Two of the most important health policies that have targeted populations without access to social security include the 1997 CCT program known as Prospera (formerly Oportunidades or Progresa) [13] and the Seguro Popular de Salud (SPS by its Spanish initials), launched in 2003 and went into operation in 2004 [14]. It is key to mention that these two programs have survived changes in the federal administrations.

CCT-Prospera has gone through four federal administration changes and SPS through three being both the most longstanding social programs targeting poor (or unprotected) populations ever in the history of modern Mexico. In the case of SPS, the General Health Law was amended to incorporate it as a permanent policy, but this is not the case of CCT-Prospera. CCT-Prospera program, seeks to improve the provision and quality of basic social services (health, nutrition, and education), largely among the most economically and socially disadvantaged populations [15]. A collaboration agreement was signed in 2009 by public health institutions in the country in order to provide health services through their medical units to all pregnant women presenting an obstetric emergency aiming at reducing maternal and perinatal morbidity and mortality [16]. Also, the official norm NOM-007-SSA2 launched in 1993, makes antenatal care mandatory in all health units. The norm had an update in 2016 [17]. However, the supply side was not the only problem, as on the demand side, the low participation of pregnant women motivated the CCT-Prospera program on maternal health services. From its inception, Prospera has operated based on a lifeline perspective and through the introduction of demand-side incentives, such as the delivery of CCT to its beneficiaries. The program offers monetary support and scholarships in cash with a bimonthly frequency provided individually to the program’s beneficiaries [15,18]. In particular, regarding reproductive health services, women enrolled in Prospera receive reproductive health consultations and health talks or “*pláticas”*, at MoH’s units, in which women are informed about family planning, pregnancy, delivery, postpartum care, and other reproductive and child health topics. Different evaluations of Prospera have shown that the program has increased the number of antenatal care (ANC) visits among its beneficiaries [15], has contributed to reduce infant mortality rates [19,20], and has improved the adequacy ANC and postnatal provide by trained personnel [19,21,22]. However, problems in access to health services persist, supply and demand for maternal health services in Mexico remain misaligned and this is aggravated mainly among the most disadvantaged communities [22-24].

It is important to note that the measurement of EC in a health system such as the Mexican one, requires considering its structural segmentation - which represents a determinant of its heterogeneous performance. In Mexico, formal workers and their families have the right to access to social security institutions by contributing a portion of their salary. Social security institutions do not receive care through service packages. In contrast, those without social security receive attention from the Ministry of Health (MoH) and other public institutions. The MoH’s service structure is decentralized and with the creation of the SPS they were provided with fresh financial resources which they had historically lacked. The SPS is voluntary and establishes financial allocation mechanisms in order to ensure that available funds are used for the direct care of users based on a package of primary and secondary care services, in which maternal health interventions stands out, plus a restricted package of high cost interventions in order to maintain financial balance and an appropriate level of cost-effectiveness.

SPS seeks to guarantee the exercise of the universal right to health through facilitating and incentivizing effective access to quality health services and promoting demand of services among the population without social security. Specifically, SPS works to achieve this ambitious goal through at least four mechanisms: 1) guaranteeing a package of health interventions stipulated in the Health Services Universal Catalogue (known as CAUSES); 2) strengthening human resource and material capacities in health clinics, with a focus on first level of care, through the incremental increase of financial resources [11,14,25,26]; 3) promoting professional care of the mother-child binomial in pregnancy, childbirth, and postpartum [26]; and 4) achieving synergies with specific programs in maternal and child health [26]. The allocation of fresh funds by SPS has been shown to increase the utilization and access to maternal health services [27-29]. However, great challenges have been pointed out in the implementation of this policy, including: (1) the need for improved administrative adherence to a legal framework; (2) ensuring financial sustainability and promoting the allocation of more resources for the health system; (3) maintaining the affiliate registry with no duplication; (4) increasing access to health care; and (5) improving quality of health care [30,31].

In previous studies, SPS has not been shown to alter the probability of receiving opportune prenatal attention [29] and differences in access to prenatal care seem to differ based on insurance status [3]. Recently, we showed population-level results that suggest, from a perspective of continuity of maternal health care [2], that Mexico is located in a sub-optimal position regarding maternal health care. According to this research, this situation could be addressed through the development of supply-demand side combined interventions, in order to achieve UHC and SDG 3.7 y 3.8 [32].

Based in these elements and following our previous research [1-3], in this study we estimate the EC of interventions aimed at improving maternal and child health in Mexico by health insurance status and test the existence of positive synergies by combining supply-demand interventions in reducing existing gaps in health insurance coverage in Mexico. We hypothesized that the observed disadvantage among SPS women compared to women with social security could be boosted by CCT-Prospera program.

## METHODS

### Settings

A retrospective cohort analysis was performed. Analysed data came from the cross-sectional Mexican National Health and Nutrition Survey conducted in 2012 (ENSANUT for its Spanish initials). ENSANUT followed a multistage, stratified design, population-based (N = 115 170 278) and representative of rural/urban strata, encompassing the 32 Mexican states and specific population groups (such as children, adolescents and adults). ENSANUT aimed to estimate the prevalence and proportions of health and nutrition conditions, access to services, health determinants, as well as coverage of health care services among the Mexican population. The ENSANUT’s response rate was close to 90% [33]. The data for analysis was requested and obtained from the survey’s public repository hosted in http://ensanut.insp.mx/. Ethical and research considerations about this survey have been previously documented [33].

For this analysis, we used data from the survey’s reproductive health module, which had been applied to a random subsample of women aged 12-49 years (n = 23 056). From these, we selected women who had their last live birth from 2005 onwards, being covered by social security, SPS or without any health insurance, and who responded to a series of questions about their use of ANC and obstetric services (n = 7144). After excluding women without information on relevant covariables (10.2%), the final analytical sample included 6413 women (N = 9 093 785). We examined potential differences in important sociodemographic and health-related characteristics that could be associated with EC between our analytical sample and those excluded due to missing data or without significant differences.

### Measures

Our main outcome is the Effective Coverage (EC) of maternal and child health care. According to previous studies [6,34], EC allows knowing “*the fraction of potential gain in health that a health system can provide through an available intervention*”. EC is a health metric that relates coverage, utilization, and access to health services. EC in maternal and child health involved four key steps: defining measures of need, identifying health interventions, use and quality. Mathematically, EC is defined as: *Q_ij_U_ij_|*(*N_ij_=1*) where *Q_ij_* is the proportion of potential health gain that is achieved from the intervention *j* received for a women *i*, *U_ij_* refers to receive the intervention conditional on need *N_ij_*.

*N_ij_* was approached by the self-reported of pregnancy among women aged 12 to 49 that reported having had an obstetric episode from 2005 to 2012 whose product was a child born alive. *U_ij_* was based in a continuity of health care approach [3] and defined a full reception of ANC from the product of the following five binary (1/0): (i) skilled ANC; (ii) timely (initial ANC visit during the first trimester of pregnancy); (iii) frequent (at least four ANC visits during the pregnancy); (iv) adequate content of ANC that include eight procedures measured in the survey according to official guidelines [17]; and (v) two indicators related to the delivery process and postnatal care (institutional and skilled delivery). In line with our previous work [3,35], all ANC procedures were weighted equally. For the definition of these indicators, our conceptual approach was based on the continuum of maternal and childcare framework, a key strategy of intervention programs for improving the health and well-being of mothers and newborns. This approach establishes that continuity of care (CoC) follows a path or route from pregnancy, to childbirth, to postpartum, where each step adds value to ensure better health outcomes for mothers and newborns and contributes to the reduction of maternal and neonatal mortality [36-38].

Finally, health gains or quality of care (*Q_ij_*) was approached by two binary (1/0) variables: no maternal complication during childbirth and the normal birth weight (in kg), measured by reviewing the official certificate or self-reported by the mother or guardian of the child. The external validity of the birth weight values collected by the ENSANUT 2012 has been previously proved [35]. We calculate the *EC* as the product of *Q_ij_*, *U_ij_* and *N_ij_*.

Our key independent variables were the self-reported health insurance condition (social security -employment based insurance-, SPS-health insurance for the poor- and none) and an indicator for being a part of a beneficiary household of the CCT Prospera program.

Other covariates included: women schooling (years), indigenous status, a standardized asset and housing index as a measure of socioeconomic status based on assets and household infrastructure, developed using polychoric correlation matrices [39], where more positive scores indicate a greater number of assets and better housing conditions, while lower socioeconomic status households have more negative scores; the type of the locality (metropolitan/urban or rural); and a social deprivation index of the place of residence (based on locality level access to basic public services, housing conditions and wage earnings). We also included maternal characteristics at the time of the most recent birth: year of the index live birth, parity, diagnosis of a health problem during pregnancy, at least one stillborn child or a child who died before the first year of life, history of abortion or miscarriage, type of delivery, frequent ANC provider (social security, the Ministry of Health-MoH- and private), and the childbirth care provider (social security, MoH and private).

### Analysis

The data were analysed using the Stata MP Package v15.1. We first describe socio-demographic and pregnancy and childbirth characteristics and each component of EC by the participation in the following women groups: social security, SPS + CCT, SPS or CCT and without health insurance + CCT. We used bivariate regression models and bivariate χ2 test to compare the groups. In order to identify correlates of the EC, we estimated the following logistic multiple regression model:


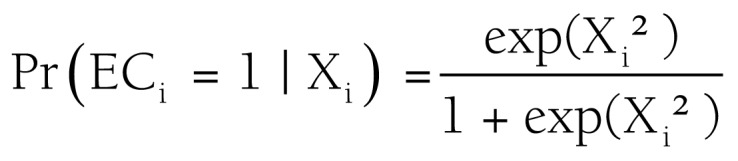


Where X_i_ was a vector containing socio-demographic and household characteristics for each *i* women, as well as covariates related to the residence context; characteristics of the respondents that related to her pregnancy history and the prenatal period and the pregnancy and delivery care indicators mentioned above. β was a vector of parameters to estimate.

This model was adjusted using maximum likelihood approach including robust standard errors and fixed effects at municipality level. For ease of interpretation we calculated adjusted odds ratios (aORs) and the corresponding 95%CI. The aOR represents the adjusted odds that an EC will occur given any specific exposure (ie, SPS + CCT), compared to the odds of the outcome occurring in the absence of that exposure. After the estimated model we adjust EC across each *i* women with social security, SPS + CCT, SPS or CCT and without health insurance + CCT, and by specific socio-demographic characteristics such as schooling, age at time of the most recent childbirth, indigenous status, socioeconomic status and deprivation level of locality, and by pregnancy and childbirth characteristics such as the number of children at time of the most recent childbirth, diagnosed with some health problem during pregnancy and type of delivery.

## RESULTS

**Table 1** shows the characteristics of the sample population by health insurance status. In comparison with individuals without social security (SPS and without health insurance), those with social security had more years of schooling (10.8), a higher age at the time of the most recent childbirth (27.1 years), less prone to belong to an indigenous household (4.7%), higher assets and housing index, lower deprivation index at the locality level, and more likely to dwell in urban areas (83.5%). In relation to pregnancy and childbirth characteristics, individuals with social security had slightly more probability of being diagnosed with a health condition during pregnancy (60.2%), and of having at least one miscarriage or abortion episode (18.9%). This group had also the lowest probability of vaginal delivery (48.7%). In terms of antenatal and childbirth care, individuals with SPS were the least likely to make use of private services (12.2% and 11.5%), and an important proportion of individuals without health insurance reported having made use of MoH services for antenatal and childbirth care (46.2% and 47.0%).

**Table 1:** Main socio-demographic, pregnancy and childbirth characteristics (mean or %, 95% CI) of the population studied

	With Social Security	With SPS and CCT-Prospera	Only with SPS or CCT- Prospera	Without health insurance and CCT- Prospera
	**(n = 1902; 29.7%)**	**(n = 1293; 20.2%)**	**(n = 2339; 36.5%)**	**(n = 879; 13.7%)**
**PANEL A: Socio-demographic characteristics:**
Schooling (years)*	10.8 (10.6-10.9)	6.8 (6.7-7.0)	8.3 (8.2-8.4)	9.1 (8.9-9.4)
Age at time of the most recent childbirth*	27.1 (26.8-27.4)	27.3 (26.9-27.7)	24.3 (24.0-24.5)	24.5 (24.1-25.0)
Indigenous*	4.7 (3.8-5.7)	22.7 (20.4-24.9)	8.7 (7.5-9.8)	7.7 (6.0-9.5)
Socioeconomic index (SD)*	0.5 (0.5-0.6)	-0.6 (-0.7–0.6)	-0.1 (-0.2–0.1)	0.1 (0.1-0.2)
Residence context – Urban/metropolitan (≥2500 inhabitants)*	83.5 (81.9-85.2)	40.9 (38.2-43.6)	65.8 (63.8-67.7)	79.2 (76.5-81.9)
Deprivation index of residence context (SD)*	-0.5 (-0.5–0.5)	0.8 (0.7-0.8)	0.1 (0.0-0.1)	-0.2 (-0.3–0.2)
**PANEL B: Pregnancy and childbirth characteristics:**
Year of obstetric episode – 2005 to 2006*	17.9 (16.1-19.6)	14.2 (12.3-16.1)	11.1 (9.8-12.4)	14.7 (12.3-17.1)
Year of obstetric episode – 2007 to 2009‡	50.5 (48.3-52.8)	49.4 (46.6-52.1)	45.5 (43.5-47.5)	49.1 (45.8-52.5)
Year of obstetric episode – 2010 to 2012*	31.6 (29.5-33.7)	36.4 (33.8-39.1)	43.4 (41.4-45.4)	36.2 (33.0-39.4)
Number of children at time of the most recent childbirth*	1.2 (1.2-1.3)	2.2 (2.1-2.3)	1.2 (1.2-1.3)	1.2 (1.1-1.3)
Diagnosis of some health problem during pregnancy*,§,‖	60.2 (58.0-62.4)	52.0 (49.2-54.7)	58.3 (56.3-60.3)	54.8 (51.5-58.2)
Child dead during the 1^st^ year or dead at childbirth*	4.2 (3.3-5.1)	7.5 (6.1-9.0)	5.6 (4.7-6.6)	4.4 (3.1-5.8)
At least one miscarriage or abortion	18.9 (17.1-20.6)	16.6 (14.6-18.7)	16.1 (14.6-17.6)	15.6 (13.2-18.1)
Vaginal delivery*	48.7 (46.4-50.9)	65.9 (63.3-68.5)	58.8 (56.8-60.8)	51.3 (48.0-54.7)
Urgent cesarean-section	26.5 (24.5-28.4)	22.8 (20.5-25.1)	25.5 (23.7-27.3)	25.1 (22.2-28.0)
Planned cesarean-section*	24.9 (22.9-26.8)	11.3 (9.6-13.0)	15.7 (14.2-17.2)	23.6 (20.7-26.4)
Frequent ANC provider – Social Security*	65.6 (63.4-67.7)	11.8 (10.0-13.5)	11.4 (10.1-12.7)	18.0 (15.4-20.5)
Frequent ANC provider – Ministry of Health*	13.3 (11.8-14.8)	83.7 (81.6-85.7)	71.8 (69.9-73.6)	43.2 (39.9-46.5)
Frequent ANC provider – Private*	21.1 (19.3-22.9)	4.6 (3.4-5.7)	16.9 (15.3-18.4)	38.9 (35.6-42.1)
Childbirth care provider – Social Security*	59.7 (57.5-61.9)	12.6 (10.8-14.5)	12.2 (10.9-13.6)	20.3 (17.6-23.0)
Childbirth care provider – Ministry of Health*	23.9 (22.0-25.8)	80.2 (78.0-82.4)	73.4 (71.6-75.2)	44.7 (41.4-48.0)
Childbirth care provider – Private*	16.4 (14.7-18.1)	7.1 (5.7-8.6)	14.3 (12.9-15.8)	35.0 (31.8-38.2)

CI – confidence interval, SPS – Seguro Popular de Salud, CCT – conditional cash transfer, ANC – antenatal care, SD – standard deviation

*P < 0.001.

†*P* < 0.01.

‡*P* < 0.05.

§Includes high blood pressure, vaginal bleeding, threat of miscarriage, preeclampsia or eclampsia, gestational diabetes, or infections.

‖Reported characteristics for 6312 women who received prenatal care.

Descriptive statistics for five process indicators and two health outcomes among the three comparison groups are shown in **Table 2**. We showed the unadjusted probabilities of occurrence for each of the outcomes. We observed that individuals with social security outperformed their counterparts in the following indicators: medical (99.2%), timely (89.4%) and frequent ANC (87.1%), and adequate content of ANC (77.5%). Concerning institutional delivery, nearly all surveyed individuals reported delivery by medical staff. Roughly, 60% of women reported no complications during childbirth among those with social security in comparison with women without social security – of which only 50% reported no complications. We also observed significant differences in the percentage of newborns with normal weight. Among women with social security almost 60% of newborns had normal birth weight whereas less than 50% of newborns on the other groups had normal birth weight.

**Table 2:** Components of effective maternal-child health coverage: unadjusted estimates of conditional coverages (mean or %, 95% CI)

	Social Security	With SPS and CCT- Prospera	Only with SPS or CCT- Prospera	Without health insurance and CCT- Prospera
**Need (*N*)**	**(n = 1902; 29.7%)**	**(n = 1293; 20.2%)**	**(n = 2339; 36.5%)**	**(n = 879; 13.7%)**
**Utilization (*U*):**
Coverage of skilled health care during ANC, %*	99.2 (98.7-99.6)	98.5 (97.9-99.2)	98.1 (97.6-98.7)	97.5 (96.5-98.5)
Coverage of timely ANC, %*,§	89.4 (88.0-90.8)	80.0 (77.9-82.2)	78.8 (77.2-80.5)	79.6 (77.0-82.3)
Coverage of frequent ANC, %*,‖	87.1 (85.6-88.6)	77.3 (75.1-79.6)	75.7 (74.0-77.5)	75.1 (72.2-77.9)
Coverage of adequate content of ANC, %*,¶	77.5 (75.7-79.4)	65.3 (62.7-67.9)	64.7 (62.7-66.6)	63.9 (60.8-67.1)
Coverage of institutional delivery, %*	77.4 (75.6-79.3)	65.1 (62.5-67.7)	64.6 (62.6-66.5)	63.6 (60.4-66.8)
**Quality (*Q*):**
Women without complication during childbirth, %*,**	63.5 (61.3-65.6)	52.7 (49.9-55.4)	51.8 (49.8-53.8)	51.5 (48.2-54.8)
Percentage of newborns with normal birth weight, %*,††	57.8 (55.6-60.0)	47.3 (44.5-50.0)	45.8 (43.8-47.9)	45.2 (41.9-48.5)

CI – confidence interval, SPS – Seguro Popular de Salud, ANC – antenatal care, CCT – conditional cash transfer

*P < 0.001.

†*P* < 0.01.

‡*P* < 0.05.

§1^st^ ANC visit during the first quarter.

‖At least four consultations.

¶≥75% of content of ANC consultation (include measurement of height, weight, and blood pressure, urinalysis, blood examination, blood glucose examination, syphilis detection-VDRL-, ultrasound, tetanus vaccine, folic acid, vitamins/iron/food supplement.

**Include preeclampsia or eclampsia, hemorrhage, miscarriage, threat of miscarriage, obstructed delivery, wrong position of the fetus, premature childbirth, or some complication due to a previous disease.

††Normal birth weight: 2.5-4.0 kg.

**Table 3** shows the aORs for EC considering exposure groups, socio-demographic, pregnancy and birth characteristics, and the locality where the mother resides. We observed that women who are beneficiaries of SPS or CCT only, as well as those without SPS and CCT, have a lower possibility of receiving EC, with respect to women who do have social security. In the case of women who received benefits from SPS and CCT we did not observe any significant difference with those with social security. Regarding the years of study, we observed that only those women with 12 years or more of education increased their chances of receiving EC with respect to women with 6 years or less. Similarly, we found that women who were 29 or older at the birth of their last child have a greater chance of receiving EC, with respect to those who were 19 years old or younger. Compared with vaginal delivery, the planned cesarean is also a factor that increases the possibility of EC. On the other hand, factors such as having two or more children, being diagnosed with a health problem during pregnancy and presenting an emergency cesarean, decrease the possibility of EC in pregnancy care. We did not observe any differences associated with the mother’s residence.

**Table 3:** Adjusted odds ratios (and robust 95% confidence intervals) from regression analyses identifying correlates for effective maternal-child health care coverage

	aOR	robust CI9 5%
**Exposure variable:**		
Social Security	Ref.	
Seguro Popular de Salud and CCT- Prospera	0.99	0.79-1.25
Only Seguro Popular de Salud or CCT- Prospera	**0.74^†^**	**0.61-0.89**
Without health insurance and CCT- Prospera	0.67*	**0.54-0.82**
**Sociodemographics:**		
Schooling – 0 to 6 years	Ref.	
Schooling – 6 to 8 years	1.12	0.90-1.40
Schooling – 9 to 11 years	1.12	0.90-1.38
Schooling – 12 or more years	**1.39^†^**	**1.09-1.77**
Age at time of the most recent childbirth – 12 to 19 years	Ref.	
Age at time of the most recent childbirth – 19 to 24 years	1.12	0.93-1.35
Age at time of the most recent childbirth – 24 to 29 years	1.11	0.90-1.38
Age at time of the most recent childbirth – 29 or more years	**1.25^‡^**	**1.00-1.55**
Indigenous	0.92	0.70-1.22
Socioeconomic level – Lowest	Ref.	
Socioeconomic level – Moderate	1.01	0.86-1.19
Socioeconomic level – Highest	1.15	0.96-1.39
**Pregnancy and childbirth characteristics:**		
Year of obstetric episode – 2005 to 2006	Ref.	
Year of obstetric episode – 2007 to 2009	1.06	0.89-1.26
Year of obstetric episode – 2010 to 2012	1.09	0.91-1.31
Number of children at time of the most recent childbirth – Zero	Ref.	
Number of children at time of the most recent childbirth – One	0.99	0.84-1.17
Number of children at time of the most recent childbirth – Two or more	**0.77^†^**	**0.64-0.93**
Diagnosed with some health problem during pregnancy	0.75*	**0.67-0.85**
Type of delivery – Vaginal delivery	Ref.	
Type of delivery – Urgent cesarean-section	0.34*	**0.30-0.40**
Type of delivery – Planned cesarean-section	**1.19^‡^**	**1.01-1.40**
Frequent ANC provider – Social Security	Ref.	
Frequent ANC provider – Ministry of Health	0.81	0.63-1.03
Frequent ANC provider – Private	0.96	0.74-1.23
Childbirth care provider – Social Security	Ref.	
Childbirth care provider – Ministry of Health	0.98	0.78-1.22
Childbirth care provider – Private	0.99	0.76-1.30
**Residence context:**		
Deprivation level – Lowest	Ref.	
Deprivation level – Moderate	0.94	0.72-1.22
Deprivation level – Highest	0.99	0.72-1.37
Urban or metropolitan	Ref.	
Rural	0.89	0.71-1.11
Constant	**1.88^‡^**	**1.07-3.30**
Observations		**6,312**
McFadden’s R^2^		0.11
Log likelihood		-3,672.93
AIC		1.48
Hosmer-Lemeshow χ^2^ (*P* > χ^2^)		15.30 (0.05)
**Link test for model specification:**		
hat (*P* > |z|)		0.00
hatsq (*P* > |z|)		0.37
Area under the ROC curve		**0.72**

SPS – Seguro Popular de Salud, aOR – adjusted odds ratio, CI – confidence interval, CCT – conditional cash transfer, AIC – Akaike information criterion

*P < 0.001.

†*P* < 0.01.

‡*P* < 0.05.The difference in the number of observations between **Table 1** and **Table 2** is due to the responses about the place of frequent prenatal care and the detection of health problems during pregnancy were obtained among women who received prenatal care.

**Table 4** shows the adjusted estimations of maternal and child effective coverage among our comparison groups. In panel A, we show socio-demographic characteristics and panel B shows pregnancy and childbirth characteristics. We observed that our global estimate of EC shows no differences between women receiving both programs – SPS + Prospera – and those with social security for each comparison group. This finding suggests that the combination of these two interventions can reduce the gap in EC between these groups. In addition, we observed that the adjusted estimates for these two groups maintain a statistically significant difference with respect to the other two most vulnerable groups. The adjusted estimations for women with only SPS or CCT did not result statistically different from those who do not have any of these benefits. This suggests that in terms of EC of maternal health services we can only observe a relevant change through the synergy of SPS and CCT.

**Table 4:** Adjusted estimations (adjusted effective coverage and robust 95% CI) of effective maternal-child health care coverage among specific women groups

	Social Security	With SPS and CCT-Prospera	Only with SPS or CCT-Prospera	Without health insurance and CCT-Prospera
**GLOBAL ESTIMATES**	54.1 (51.3-56.9)	53.9 (50.6-57.2)	47.6 (45.4-49.8)	45.5 (42.1-48.9)
**PANEL A: Socio-demographic characteristics:**				
Schooling (years):				
0 to 6	50.8 (45.9-55.7)	50.6 (45.6-55.7)	44.2 (39.8-48.7)	42.2 (37.1-47.3)
12 or more	57.8 (54.3-61.2)	57.6 (53.4-61.9)	51.3 (47.8-54.8)	49.2 (44.9-53.5)
Age at time of the most recent childbirth (years):				
12 to 19	51.4 (47.0-55.7)	51.2 (46.5-55.9)	44.8 (41.0-48.7)	42.8 (38.3-47.3)
29 or more	56.1 (52.7-59.5)	55.9 (52.2-59.7)	49.6 (46.5-52.7)	47.5 (43.4-51.6)
Indigenous status:				
Non-indigenous	54.3 (51.4-57.1)	54.1 (50.8-57.5)	47.7 (45.5-50.0)	45.7 (42.2-49.1)
Indigenous	52.6 (46.5-58.7)	52.4 (46.1-58.8)	46.0 (40.1-52.0)	44.0 (37.6-50.4)
Socioeconomic level:				
Lowest	53.0 (49.2-56.7)	52.8 (48.9-56.7)	46.4 (43.3-49.5)	44.3 (40.3-48.4)
Highest	56.0 (52.8-59.3)	55.9 (51.8-59.9)	49.5 (46.4-52.6)	47.4 (43.4-51.5)
Deprivation level of residence context:				
Lowest	55.9 (51.6-60.2)	55.7 (51.1-60.3)	49.4 (45.3-53.4)	47.3 (42.4-52.2)
Highest	53.3 (50.2-56.5)	53.2 (49.5-56.8)	46.8 (44.2-49.4)	44.7 (41.1-48.4)
**PANEL B: Pregnancy and childbirth characteristics:**				
Number of children at time of the most recent childbirth:				
Zero	56.3 (52.7-59.9)	56.2 (52.0-60.3)	49.8 (46.5-53.0)	47.7 (43.5-51.9)
One	56.2 (52.9-59.5)	56.0 (52.2-59.9)	49.7 (46.7-52.6)	47.6 (43.7-51.6)
Two or more	50.8 (47.4-54.3)	50.7 (47.0-54.4)	44.3 (41.3-47.2)	42.3 (38.4-46.1)
Diagnosed with some health problem during pregnancy	51.5 (48.5-54.5)	51.4 (47.8-54.9)	45.0 (42.5-47.4)	42.9 (39.3-46.5)
Type of delivery:
Vaginal delivery	59.4 (56.3-62.4)	59.2 (55.7-62.7)	52.6 (50.1-55.2)	50.5 (46.7-54.3)
Urgent cesarean-section	35.9 (32.5-39.4)	35.8 (31.9-39.7)	29.9 (27.0-32.7)	28.1 (24.5-31.6)
Planned cesarean-section	63.0 (59.2-66.8)	62.9 (58.6-67.1)	56.4 (52.8-60.0)	54.3 (49.8-58.8)

SPS – Seguro Popular de Salud, CCT – conditional cash transfer, CI – confidence interval

## DISCUSSION

We corroborate our hypothesis that the complementary use of these interventions can reduce the gaps in EC for maternal health services among population groups with specific characteristics of social vulnerability.

First, adjusted estimates of EC suggest that overall, having both programs – Prospera + SPS – has a positive effect on maternal and child care coverage, which makes the observed differences in EC not statistically significant between those affiliated to both programs in comparison with the observed in the population with social security. It is important to note that EC in the social security system is not the ideal and that there are many areas of improvement in these institutions. However, they represent the best possible value of EC in the country and therefore a point of national reference. Second, the participation to only one of the programs or not being affiliated to any health insurance was associated with a significant reduction on the coverage of maternal care, in comparison with the other groups analyzed. Our findings are consistent with previous studies that have shown that the combination of strategies from the supply and demand sides increases the likelihood of using health services [10]; however, they also mentioned that even when there are high levels of access to primary health care, other determinants of health services utilization must be addressed particularly for lower-income populations [8,10]. A woman who is a Prospera beneficiary has to compulsorily attend antenatal consultations, however, as our results suggest, Prospera by itself cannot guarantee the utilization of those services as it only induces the demand of these services calling from a timely response from a supply-side program (SPS) to increase their utilization. There is also the same gap in the use of health care services among women only covered by SPS, suggesting a lack of communication and dissemination campaigns that promote and encourage the use of maternal and child health care services.

We also identified population subgroups for which being affiliated or not to the programs analyzed did not make a difference in the EC of maternal and childcare services. This is the case for women with fewer years of schooling (0-6 years.), adolescents, and indigenous women, which is consistent with the widely evidenced association between mother education and child health [8]. In these population subgroups, no significant differences were found between the four scenarios evaluated (with social security, with Prospera + SPS, with Prospera or SPS and without Prospera nor health insurance). This reflects that for the most vulnerable groups the benefits of the analyzed programs are not enough to achieve significant changes in the EC of maternal and child health services and that more comprehensive policy efforts are needed to revert these indicators, generate greater equity and obtain better results in maternal and child health outcomes. Additionally, subgroups where only the combination of both programs equals the coverage of maternal health services to those observed in the population with social security. This is the case of women 29 years of age or older, non-indigenous women, those who live in households with a higher score in the asset index, those who live in highly marginalized areas, and those diagnosed with complications during pregnancy. Particular attention should be paid for those subgroups of population from urban areas with low socioeconomic status, without health insurance and without any access to other type of social programs such as Prospera. That is the case of young women who are not having any support from the demand side, through the access to social programs, focus on improving the adequate use of reproductive health services of young women, or some support from the supply side, through the coverage of a health insurance. The combination of both strategies could increase the chances to approach effective reproductive health services by these subgroups.

Previous studies have documented the effects of supply and demand-side interventions and showed an increase in the use of health care services and in the reduction of child mortality, mortality among children 5 years of age and under and maternal mortality [8,9]. However, they highlight to pay special attention to the organizational, logistic, and administrative aspects from the supply side of health services. For example, attention should be paid to avoid corrupt practices and lack of transparency that reduce the possibility that these financing schemes really have an impact on the reduction of direct out-of-pocket spending by users and that contribute to a loss of confidence in the way these interventions are implemented. Another aspect to be considered and that has been documented, is the need to increase efficiency in the use of resources. Inefficiency in the provision of health services is another element that puts at risk the possibility of achieving the objectives proposed by these interventions. In this respect, we have recently shown that the steady escalation of ﬁnancial resources in the public health subsystem over the past 15 years has yielded sub-optimal results as regards coverage for essential maternal health interventions among the poorest; suggesting that the Mexican government must put in place a set of measures to guarantee efﬁciency in the system’s performance without affecting equity gains [40].

A key element to take into consideration to adequately weight the value of these programs and their joint positive effect is the political use that have been made of them. On the SPS side, inefficiencies of allocation, inexperienced management, and diversion of resources by local political actors have been documented [41,42]. These factors reduce the capacity of the program to maximize achievements in the production of services, the reduction of out-of-pocket expenditure, and the improvement of health conditions. In the case of Prospera improper use of funds for the unjustified purchase of goods and services, and the allocation of subsidies to promote the vote in favor of the party in the government have been observed. The shielding of these programs against these types of abuse and deviations could raise the possibility of increasing the positive effect shown by the study.

We show that some factors such as years of study (12 or more), age (being 29 or older), and having a planned cesarean are factors associated with higher EC. Also, these factors might be highly associated with more information, empowerment and, autonomy of the mother. For this reason, in the case of policies from the demand side, it is necessary to strengthen the communication mechanisms in the communities, increasing the maternal knowledge of health, and empowerment and autonomy of women to increase the use of maternal and child health services, through communication and dissemination campaigns about adequate health services to expect better care, as well as social support to ask for a better care, and maximize the potential benefits in those population groups with the greatest need. More information about their rights and the benefits of these interventions improves the population empowerment and their ability to benefit from these policy initiatives.

There are some limitations to our study. First, although ENSANUT is a high-quality, population-based survey, our study shares the limitations of any study based on self-reported data; therefore, we only include births in the past 5 years to reduce the probability of recall bias. Second, this is a cross-sectional study and we report associations, not causal effects. Third, although it would be ideal to rely on clinical data to evaluate receipt and content of ANC, obstetric outcomes and birth weight; however, and unfortunately in Mexico such a data set does not exist. Fourth, it is important to highlight that our analysis uses an indicator that allows us to approximate not only the use of services when there is a need (in this case pregnancy), but also includes an element that allows us to measure the gain in health through the reception of a quality service (according to official norms) during the maternal care continuum. Previous studies have also identified that for these interventions to be successful, they must be offered with quality, in order to cover the expectations of the users and be based on respectful care [8]. The analytical approach based on the continuity of care could help to dimension and assess more objectively the performance of the Mexican health system, as well as identify the “bottlenecks” in the stages of maternal care and the scope of the various policy initiatives implemented. Finally, we did not fail to mention that the participation in the social programs included in our study (Prospera and SPS) could be limited since it is based on self-report of the participants and not on administrative records. Although the self-report could have errors, it has been also documented problems of incorporation into the SPS [43] or that its beneficiaries are not correctly classified according to their socio-economic condition [44]. In this sense, it is difficult to establish what the impact of this situation would be on the estimates presented. However, in favor of our study, we can mention that several studies have proved the close relationship between living conditions and self-report of belonging to the social programs analyzed or social security [45-47].

## CONCLUSIONS

Our results suggest that: i) the synergistic effect in the combination of coverage for the SPS and Prospera, is associated with levels of EC of maternal health services similar to those observed in the population with social security coverage; ii) the most vulnerable population groups need to be reached by the combination of both programs, so that public health policy efforts are reflected in greater EC of maternal health services and better maternal and child outcomes. Special attention must be offered for the most vulnerable groups (women with fewer years of schooling, adolescents, and indigenous women), for whom the benefits of the analyzed programs are not enough to achieve significant changes in the EC of maternal and child health services. These results identify potential groups in which the gains from directing programs that increase the EC of maternal health services can be cost-effective and highlight gaps in the performance of the Mexican health system and identified several gaps in the cascade of the maternal-child care. Our study also provides evidence that allows us to measure the potential gains of more comprehensive policy efforts needed to revert these indicators and generate greater equity. These results are relevant given the major changes that the Mexican Health System is undergoing, and the redefinition of the strategies that the Mexican government is undertaking to achieve universal and effective health coverage.
